# Corrosion Risk Assessment in Coastal Environments Using Machine Learning-Based Predictive Models

**DOI:** 10.3390/s25134231

**Published:** 2025-07-07

**Authors:** Marta Terrados-Cristos, Marina Diaz-Piloneta, Francisco Ortega-Fernández, Gemma Marta Martinez-Huerta, José Valeriano Alvarez-Cabal

**Affiliations:** Project Engineering Department, University of Oviedo, 33004 Oviedo, Spain

**Keywords:** corrosion monitoring, atmospheric corrosion, machine learning, chloride deposition, life assessment, predictive modeling, coastal infrastructure

## Abstract

**Highlights:**

**What are the main findings?**

- A machine-learning-based framework using gradient boosting, SVM, and neural networks accurately predicts chloride deposition in coastal environments using publicly available environmental variables.
- Gradient boosting outperformed other models, achieving an F1 score of 0.8673 and an AUC of 0.95, with key predictors identified as land coverage, wind, and relative orientation.

**What is the implication of the main finding?**

- The proposed approach enables an early-stage corrosion risk assessment without the need for long-term monitoring, supporting more efficient and sustainable design in coastal infrastructure.
- By aligning model outputs with ISO 9223:2012 standards, this method offers a practical, scalable alternative for corrosion classification and structural life prediction in marine-influenced environments.

**Abstract:**

Atmospheric corrosion, especially in coastal environments, presents a major challenge for the long-term durability of metallic and concrete infrastructure due to chloride deposition from marine aerosols. With a significant portion of the global population residing in coastal zones—often associated with intense industrial activity—there is growing demand for accurate and early corrosion prediction methods. Traditional standards for assessing atmospheric corrosivity depend on long-term empirical data, limiting their usefulness during the design stage of infrastructure projects. To address this limitation, this study develops predictive models using machine-learning techniques, namely gradient boosting, support vector machine, and neural networks, to estimate chloride deposition levels based on easily accessible climatic and geographical parameters. Our models were trained on a comprehensive dataset that included variables such as land coverage, wind speed, and orientation. Among the models tested, tree-based algorithms, particularly gradient boosting, provided the highest prediction accuracy (F1 score: 0.8673). This approach not only highlights the most influential environmental variables driving chloride deposition but also offers a scalable and cost-effective solution to support corrosion monitoring and structural life assessment in coastal infrastructure.

## 1. Introduction

A large portion of the buildings and structures designed in engineering projects are metallic, typically made of steel, and are either bare or coated. Metallic structures, when exposed to outdoor environments, undergo degradation and atmospheric corrosion, requiring mitigation strategies to ensure structural safety throughout their useful life [1]. These measures include increasing the amount of steel used to compensate for material loss due to wear or adding coatings to delay material loss effects, typically employing zinc-rich alloys (galvanization) [2]. However, as with any aspect of engineering, a balance must be struck between ensuring structural integrity and managing production costs, incorporating safety factors that do not hinder project execution [3].

A particularly critical issue is the degradation of metal structures in coastal areas due to its far-reaching impact on society. Approximately 40% of the world’s population lives within 100 km of the coast [4], and these areas are often where industries in developed countries are located. For materials exposed to outdoor conditions, corrosion rates have shown linear relationships with atmospheric salt content, affecting not only metal structures but also concrete ones [5]. The effect of marine atmospheres primarily extends a few hundred meters from the coastline and decreases rapidly as one moves inland [6]. This atmospheric corrosion accelerates concrete degradation by facilitating the penetration of chloride ions into its structure. Globally, the direct costs of corrosion account for around EUR 1.3–1.4 trillion [7], highlighting the importance of efficient mitigation strategies.

The current regulations on coating metallic structures outline atmospheric corrosivity levels in ISO 9223:2012 [8]. These levels are grouped into categories established by studying the corrosion effect on standard samples during the first year of exposure to the elements. However, this classification relies on variables that are not easily accessible, requiring annual data collection on parameters not directly obtainable from meteorological variables, which complicates the availability of sufficient information for characterization.

One of these variables is precisely chloride ions, whose corrosive effect is clear, while their annual average characterization is complex since it is unrealistic to wait a year to gather data before beginning the design of a project once its location is determined. This issue reduces the usefulness of the aforementioned standards, which, while very accurate, are not readily applicable in most initial cases. This results in suboptimal designs that lean towards maximum values, adding more material than necessary and leading to economic and environmental sustainability issues due to excessive resource use.

The objective of this work is to develop a generalized model, applicable to any geographic location, for categorizing marine contaminant deposition data. This is achieved by leveraging available climatic variables and training several machine-learning models on experimental data, both from the literature and our own experiments. The ultimate goal is to characterize an environment without requiring long testing periods and sampling costs, with the aim of generalizing the results. This will significantly enhance the existing knowledge of atmospheric contaminants, specifically chloride ions, which play a crucial role in atmospheric corrosion. Consequently, structures can be sized more accurately, typically reducing or at least maintaining the need for excess thickness or coatings, thereby optimizing outcomes.

This document begins with a description of the corrosion problem in coastal regions and a state-of-the-art review focused on its relation to chloride ion pollutants, analyzing the various approaches, highlighting their limitations and the research gap addressed in this study. Following this, the methodology section details the construction of a hybrid dataset combining experimental chloride deposition measurements, meteorological variables, and data from international programs. The study evaluates the predictive performance of different supervised machine-learning models and presents model evaluation metrics. Finally, the discussion interprets the findings in the context of ISO 9223:2012 [8] corrosion classification standards, assesses the applicability of the proposed approach for structural design and maintenance, and outlines the study’s limitations and directions for future research.

## 2. Literature Review

Atmospheric corrosion is a complex electrochemical phenomenon influenced by numerous environmental factors, where the atmospheric composition plays a central role. Pollutants such as solid particles, aerosols, and gases from various sources significantly contribute to corrosion by depositing on metal surfaces. Together with humidity, temperature, orientation, and material properties, those factors modulate the corrosion rate [9,10,11]. Among these pollutants, chloride ions stand out as corrosion accelerators, especially in saline coastal regions, where marine aerosols significantly increase environmental aggressiveness [12,13,14]. While secondary sources, such as biomass combustion and industrial emissions, also contribute, ocean salinity remains the dominant factor [15,16].

The exponential decrease in the chloride concentration with increasing distance from the coast has been well-documented since 1960, with significant deposition observed within 100 km of the ocean [17]. However, despite this long-established relationship, chloride deposition remains a complex phenomenon influenced by atmospheric circulation patterns, seasonal variability, and local topography [18]. To better understand these dynamics, recent research has incorporated predictive models that account for these additional environmental factors. Chlorides facilitate corrosion by absorbing moisture, increasing electrolyte conductivity, and altering the phases of corrosion products [10]. This complexity has led to variable parameterization of chloride deposition based on geographic and atmospheric conditions [19,20].

Several factors significantly affect chloride deposition, including distance from the coast [21], wave height [22], ocean salinity [13], elevation [23], and structural orientation [24]. Given the influence of multiple interacting variables, accurately predicting chloride deposition requires a multidisciplinary approach that integrates both empirical data and advanced modeling techniques [25]. The relationship between corrosion and salinity was first identified by Ambler and Bain in 1955 [26]. Later, in 1986, the ISOCORRAG program promoted standardization in atmospheric corrosion studies [27,28]. In 1988, the MICAT program extended this research across Ibero-American countries, allowing for the development of mathematical models to predict corrosion as a function of climate and pollutants, with a particular focus on chlorides [29]. Building on these foundational studies, more recent programs have integrated remote sensing and machine-learning techniques to enhance predictive capabilities [30].

Predictive models for corrosion can be categorized into three main types: (i) empirical models, which rely on statistical correlations but often lack generalizability beyond their initial dataset [31]; (ii) physical models, which use mathematical equations based on electrochemistry, thermodynamics, and materials science but require detailed input parameters [32]; and (iii) AI-based models, which can capture complex nonlinear interactions but demand extensive training datasets [33,34], Despite their advancements, these models differ in terms of input requirements, computational complexity, and applicability to real-world scenarios. All the studies mentioned agree that chloride deposition is essential for understanding and predicting atmospheric corrosion, especially in coastal environments. However, limitations in measurement methods and the complexity of contributing factors pose significant research challenges [35]. To provide a comprehensive overview of the chloride deposition modeling approaches, Table 1 summarizes the key methods used in previous studies.

Although these models provide valuable insights, their early-stage applicability is restricted by the need for specific input data, which is often unavailable. The high cost and time required for data collection further limit their practicality. In response, machine learning and data mining emerge as efficient alternatives, leveraging existing data to enhance predictive accuracy and streamline decision-making.

Building on previous research, the selection of input variables emerges as a crucial factor in improving chloride deposition modeling. Research conducted on the island of Oahu, Hawaii, demonstrated that proximity to the coast is the primary determinant of chloride deposition rates, with a sharp decline observed beyond 1 km from the shore [21]. On the Korean Peninsula, predictive models have highlighted the combined influence of relative humidity and temperature on chloride deposition, revealing how these factors intensify during seasonal monsoons [44]. In coastal areas of Saudi Arabia, models incorporating wind orientation and dust storms have shown that airborne salinity fluctuates significantly depending on the prevailing wind conditions [45]. Studies in New Zealand and Spain have provided further evidence of how structural positioning relative to wind direction modulates pollutant deposition, confirming that surfaces exposed to the prevailing winds experience significantly higher corrosion rates [46,47]. These findings collectively highlight the importance of selecting key input variables for predictive models. Integrating geographical, climatic, and exposure-related factors allows for a more accurate framework that aligns with real-world conditions.

Given the challenges of acquiring detailed input variables for complex models, a more accessible approach is to classify chloride deposition levels using an established standard. The ISO 9223:2012 [8] standard categorizes levels of marine aerosol contamination into four classes based on the chloride deposition rate (Table 2) [48], reflecting increasing environmental aggressiveness and corrosion risk.

These categories correspond to different levels of impact: S_0_ represents minimal corrosion, common in inland areas; S_1_ corresponds to low deposition environments requiring basic protection; S_2_ indicates moderate corrosion risks near coastlines; and S_3_ reflects highly corrosive environments, such as industrial sites near the ocean, requiring advanced protection strategies.

However, while the ISO 9223:2012 standard provides a practical classification framework, it remains a broad descriptive tool that does not incorporate site-specific environmental variability. On the other hand, more advanced predictive models offer high precision but often rely on complex input parameters that are not readily available in early-stage assessments. This creates a gap between two extremes: relying on a simplified classification system with limited input data or attempting to achieve high accuracy through models that demand extensive environmental monitoring.

To bridge this gap, there is a need for an intermediate approach that leverages easily obtainable environmental parameters, such as distance from the coastline, relative humidity, and prevailing wind conditions, to refine chloride deposition estimates beyond the categorical levels of ISO 9223:2012. By integrating empirical observations with predictive techniques, particularly machine learning, it is possible to enhance the standard classification system with more nuanced, data-driven insights without requiring highly specialized datasets. This research aims to develop such a framework, balancing accessibility and precision to improve corrosion risk assessment and decision-making in coastal environments.

## 3. Materials and Methods

### 3.1. Data

The study database combines data from international programs such as MICAT [29], localized studies [21,42], and experimental tests conducted using three sampling techniques: wet candle, dry gauze, and modified wet candle [49], according to ISO 9225:2012—Annex C [50].

**Figure 1:**
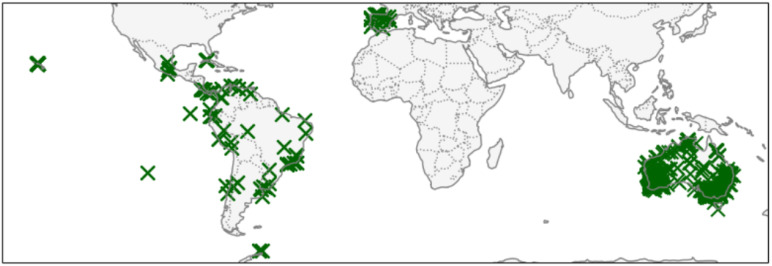
Distribution of locations for the study.

Figure 1 shows the sample distribution across the various test locations. These datasets provide specific information on chloride deposition under different conditions and geographical contexts, offering a comprehensive view of the issue under study.

Understanding the environmental drivers of chloride deposition in marine-influenced regions requires a comprehensive selection of variables capable of capturing both natural conditions and site-specific exposure characteristics. For this purpose, a diverse set of predictors was defined, informed by the established corrosion literature, and structured to reflect three analytical dimensions:

1. Spatial Context

This dimension captures the physical location and elevation of each site, specifically through:

  - Proximity to the sea, a key determinant of aerosol salinity exposure;
  - Altitude, which influences both wind dynamics and moisture retention.

2. Atmospheric Conditions

These variables describe the meteorological environment affecting corrosion potential:

  - Wind characteristics (speed and predominant direction), which govern the transport of marine aerosols;
  - Relative humidity, temperature, and precipitation, all of which contribute to the formation and persistence of moisture films on surfaces, which are crucial for corrosion initiation.

3. Exposure Configuration

To account for how environmental factors interact with site-specific features, two synthetic categorical variables were introduced:

  - Environmental coverage, ranging from sparse (open terrain) to dense (urban or forested areas), influences the shielding effect against airborne chlorides;
  - Relative orientation, reflecting the sample’s alignment with respect to pre-vailing wind flows, distinguishes between windward and sheltered (leeward) positions.

This multidimensional variable framework was applied within a supervised learning context, enabling the classification of corrosion risk levels according to ISO 9223:2012 chloride deposition thresholds. By combining physical, climatic, and situational data, the model provides a more granular understanding of how different environmental factors jointly influence corrosivity in coastal zones (Table 2).

### 3.2. Methodology

**Figure 2:**
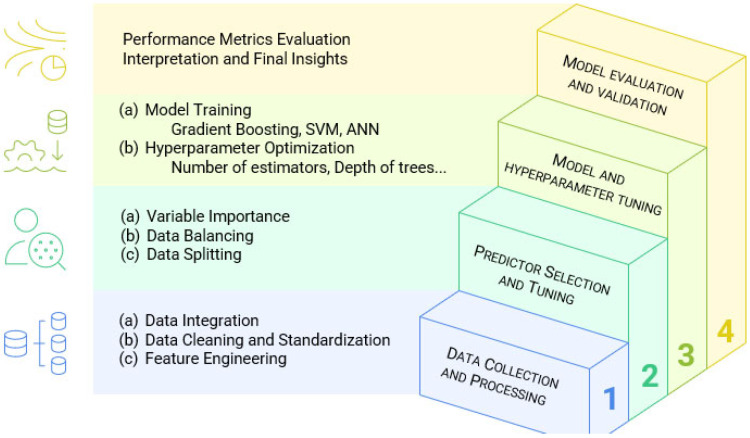
Methodological framework.

A comprehensive methodological framework was applied to develop and evaluate predictive models for chloride deposition in coastal environments (Figure 2). The process was structured in four main stages: data preparation, predictor selection, model training, and evaluation.

1. Data collection and processing: High-quality data is essential for ensuring valid and reliable model outcomes. The raw dataset underwent an extensive preparation phase. Meteorological variables, such as temperature, relative humidity, wind parameters, and precipitation, were sourced from public repositories and merged into a centralized dataset. This integration was followed by a cleaning phase, which included unit normalization, the imputation of missing values, and the removal of outliers to minimize statistical noise.
  To enrich the dataset’s explanatory power, synthetic variables were engineered. These included interaction terms (e.g., relative humidity × wind speed) and ratio-based indicators (e.g., RH/precipitation and altitude-to-distance ratio), aimed at capturing non-linear or compound environmental effects. Non-linear transformations, such as logarithmic scaling and squaring, were applied to select variables like distance and temperature to better reflect their environmental behavior and reduce skewness;
2. Variable selection and dataset structuring: The next step involved identifying the most relevant predictors for modeling chloride deposition. A random forest algorithm was employed to rank variable importance using the mean decrease in impurity (Gini index). This data-driven selection ensured that only the most informative features were retained for training. Given a pronounced imbalance in the target variable classes—where one category significantly outweighed the others—the Synthetic Minority Over-sampling Technique (SMOTE) was implemented. This technique synthetically generated new data points for the minority classes through interpolation, improving class representation and reducing model bias.
  The dataset was then partitioned into training (80%) and testing (20%) subsets. To ensure robust evaluation, five-fold cross-validation was applied. This iterative procedure filtered out inconsistencies and produced averaged performance metrics, improving the generalizability of the model;
3. Model training and hyperparameter optimization: Three supervised-learning algorithms were selected for comparison: gradient boosting (GB), artificial neural networks (ANN), and support vector machines (SVM). These models were chosen for their complementarity in capturing both linear and complex non-linear patterns.
  To maximize model performance, hyperparameters were tuned using grid search. For GB, this involved adjusting the number of estimators, tree depth, and minimum sample splits. SVMs were optimized by experimenting with different kernel functions and regularization strengths, while ANN configurations varied in terms of hidden layer architecture, activation functions, learning rates, and regularization strengths (alpha), and included early stopping to avoid overfitting. The optimization process was designed to balance prediction accuracy with computational efficiency. All models were implemented in Python 3.10 using the scikit-learn, Keras, and XGBoost libraries. To ensure reproducibility, a fixed random seed (30) was used across all training processes.
4. Model evaluation: Model performance was assessed using a combination of complementary metrics. Accuracy provided an overall measure of classification correctness, while precision and F1 score captured the model’s ability to correctly identify positive cases and balance errors. The AUC (area under the ROC curve) offered insight into the model’s ability to distinguish between classes across thresholds, and the confusion matrix revealed detailed patterns of misclassification. Together, these metrics enabled a thorough and nuanced evaluation of each model’s predictive capabilities, supporting the identification of the most effective approach for assessing the chloride deposition risk in coastal environments [51,52].

### 3.3. Techniques

In this study, various techniques were employed at different stages, with each selected for its optimal performance within its respective domain. Specifically, the Synthetic Minority Over-sampling Technique (SMOTE) was used to address class imbalance by generating synthetic samples for the minority class [53]. SMOTE has been selected because it can be particularly effective on datasets where the imbalance ratio is significant, as it avoids the problem of overfitting and helps classifiers to better capture the characteristics of the minority class [53,54]. SMOTE has proven its capability in improving the predictive accuracy of imbalanced datasets commonly found in environmental data applications [55].

With respect to modeling, a variety of techniques were employed to ensure comprehensive coverage of different approaches to prediction, specifically incorporating both tree-based and machine-learning methods.

Gradient boosting was selected as a representative of tree-based techniques due to its well-established robustness and ability to model complex, non-linear relationships. This model has demonstrated strong performance in environmental modeling tasks, particularly in handling high-dimensional data and feature interactions, leveraging the power of ensemble learning, combining multiple decision trees to reduce overfitting and improve generalization. GB refines predictive accuracy by incrementally adjusting to residual errors from previous iterations and has been shown to perform well in complex, heterogeneous datasets by constructing highly accurate trees that target difficult-to-predict data patterns [56,57]. The model’s flexibility in handling mixed data types and its superior accuracy in environmental data analysis make it a valuable tool for this study. This technique is widely used in environmental modeling and has shown superior performance in diverse prediction tasks due to its flexibility and interpretability.

Additionally, SVM and ANN were chosen to represent more advanced machine-learning techniques. Their inclusion allows for a comparison between traditional statistical-learning approaches and deep-learning-based methods, providing insights into their respective advantages in predictive accuracy and generalization. SVMs were employed to construct optimal hyperplanes that classify observations based on chloride deposition levels. This technique maximizes the margin of separation between classes, making it effective in non-linear classification tasks [58]. The ability of SVMs to handle non-linear relationships through kernel functions has proven advantageous in environmental and chemical data modeling, where interactions among variables are often complex and multi-dimensional [59].

On the other hand, ANN, as a deep-learning method, was selected to capture complex, non-linear relationships between predictor variables and chloride deposition. Such architectures have been shown to be effective in capturing the underlying patterns in environmental data [60,61]. In particular, neural networks are increasingly applied to environmental studies due to their adaptability to diverse datasets and their robustness in predictive tasks [62].

The combination of these techniques ensures that a wide range of modeling strategies are tested, allowing for a thorough evaluation of predictive performance across different algorithmic paradigms.

## 4. Results

This section outlines the results of the analysis, with a particular emphasis on the key factors influencing chloride deposition, the impact of class-balancing techniques, and the comparative performances of the predictive models evaluated.

### 4.1. Data Collection and Processing

A hybrid dataset was constructed by integrating data from the published literature, international programs, and local experimental trials. The final database comprises 449 distinct geographic locations. Several sites include multiple measurements, resulting in a total of 690 individual data entries.

**Figure 3:**
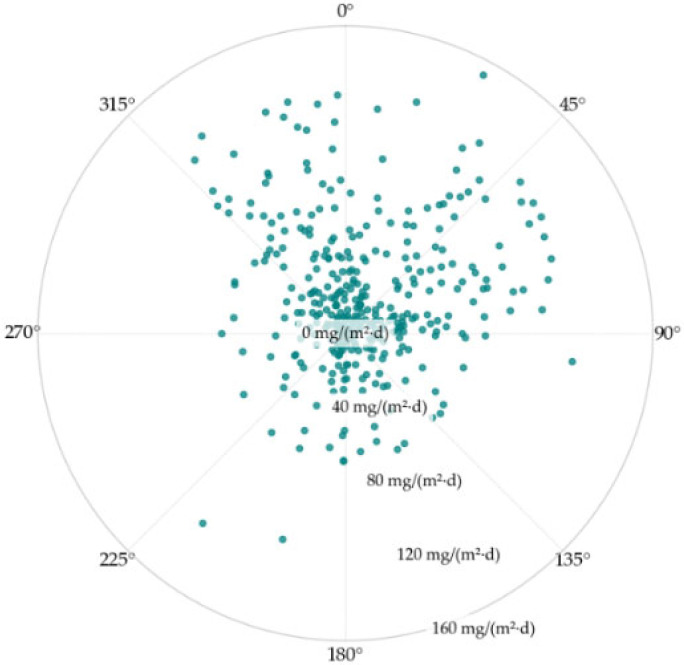
Polar graph of chloride deposition based on its angular difference between sample orientation and the prevailing wind direction (relative orientation).

The analysis of real and synthetic variables provides interesting insights into their implications and relevance. As an example, the polar graph (Figure 3) illustrates the relationship between chloride deposition and the angular difference between the sample orientation and the prevailing wind direction.

The graph shows that samples with a smaller angular difference (close to 0°), indicating alignment with the wind, exhibit higher chloride deposition levels, as reflected by their greater distance from the center of the graph. Conversely, the samples with larger angular differences show lower deposition levels.

### 4.2. Predictor Selection and Data Tuning

To analyze atmospheric corrosivity and its influence on chloride deposition, a set of key variables, including geographic, climatic, exposure-related, and synthetic, previously described, was selected. These variables were assessed to determine their predictive relevance for estimating chloride deposition levels. The variable importance analysis highlights the most influential predictors: land coverage (14%), wind speed and direction (10%), and relative orientation (8%). Additionally, synthetic variables such as the interaction between relative humidity and wind (7%) demonstrated a notable contribution to the model. The squared distance from the coastline, accounting for 7% of the variance, effectively captures the nonlinear decay in chloride deposition with an increasing distance from the sea.

To enable effective analysis, five key variables were selected as the foundation for the dataset, followed by thorough preprocessing, including data cleaning and the elimination of outliers. The original dataset contained 140 observations collected from physical corrosion experiments. Given that this volume was insufficient to support robust model training, data augmentation was performed. Moreover, the dataset exhibited a strong class imbalance: class S_1_ dominated with 71% of instances, while classes S_0_ and S_2_ accounted for only 5% and 24%, respectively. To correct this imbalance, the SMOTE algorithm (Synthetic Minority Over-sampling Technique) was implemented, generating synthetic samples for underrepresented classes by interpolating between nearby data points in feature space. This strategy helped mitigate classification bias and ensured a more equitable distribution of samples across all classes. As a result, the augmented dataset reached a total of 450 instances with balanced class representation, as detailed in Table 3.

This balanced distribution ensures that the predictive models do not favor the majority class S_1_, which enables a more accurate evaluation of their performance across all classes.

### 4.3. Modeling and Hyperparameter Optimization

The data were divided into training (80%) and testing (20%) sets, with cross-validation applied to enhance generalization and avoid overfitting. The cross-validation process was repeated five times, discarding extreme results and averaging the remaining values to ensure robust performance estimation. In this analysis, three supervised classification models, namely GB, SVM, and ANN, were studied to optimize their performance in classifying the salinity category. For each model, different combinations of hyperparameters were explored using grid search optimization. The following Table 4 summarizes the grid values for each model.

For gradient boosting, the hyperparameters optimized included the learning rate (learning_rate), the number of trees (n_estimators), and the maximum depth (max_depth). In the case of SVM, values for the regularization parameter (C), kernel type (kernel), and kernel coefficient (gamma) were explored. Finally, for the neural network model, configurations for hidden layer sizes (hidden_layer_sizes), activation function (activation), and initial learning rate (learning_rate_init) were analyzed. In addition, early_stopping and a high maximum iteration limit (max_iter = 3000) were employed to ensure convergence while avoiding overfitting.

### 4.4. Model Evaluation and Validation

After optimizing the hyperparameters, the performances of the three models were evaluated using key metrics, such as precision (positive prediction accuracy) and recall (positive detection rate). These metrics, summarized in Table 5, provide a detailed comparison of each model’s ability to correctly predict and detect positive cases.

**Figure 4:**
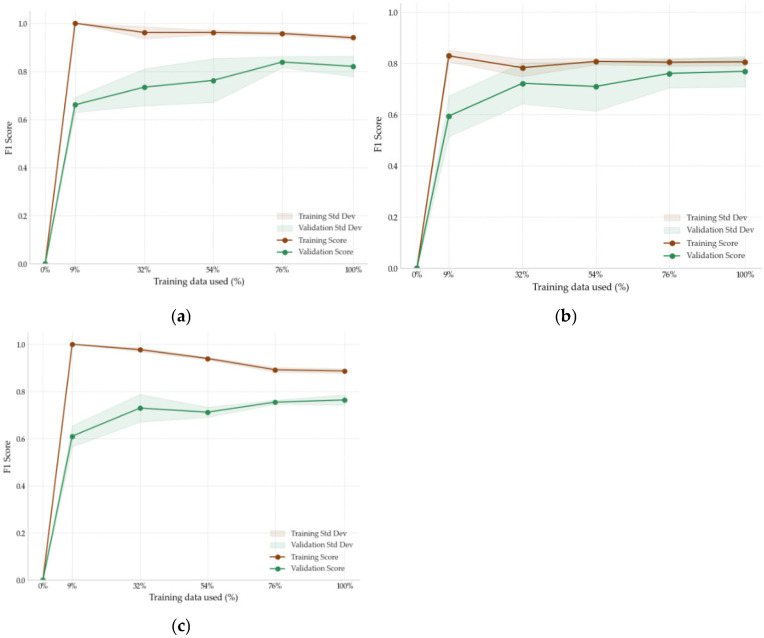
Learning curves for evaluation and optimization of: (**a**) gradient Boosting, (**b**) SVM, (**c**) neural network.

To offer a more comprehensive assessment, the F1 Score, which balances precision and recall, was also calculated. Figure 4 presents the learning curves for each model, showing how performance, measured by the F1 Score, evolves with an increasing training set size. These curves provide insights into each model’s ability to generalize to unseen data and highlight patterns of learning behavior.

- Gradient boosting: The validation curve closely followed the training curve, maintaining a stable performance with an F1 Score of 0.86 and showing fewer signs of overfitting;
- SVM: The validation curve reached an F1 Score of 0.75, remaining below the tree-based models, and did not show a noticeable gap from the training curve, suggesting that the model is more robust but less efficient;
- Neural network: The model achieved the lowest performance, with an F1 Score of 0.63. The curves indicated limited learning capacity, as the validation performance remained low despite increases in training size.

**Figure 5:**
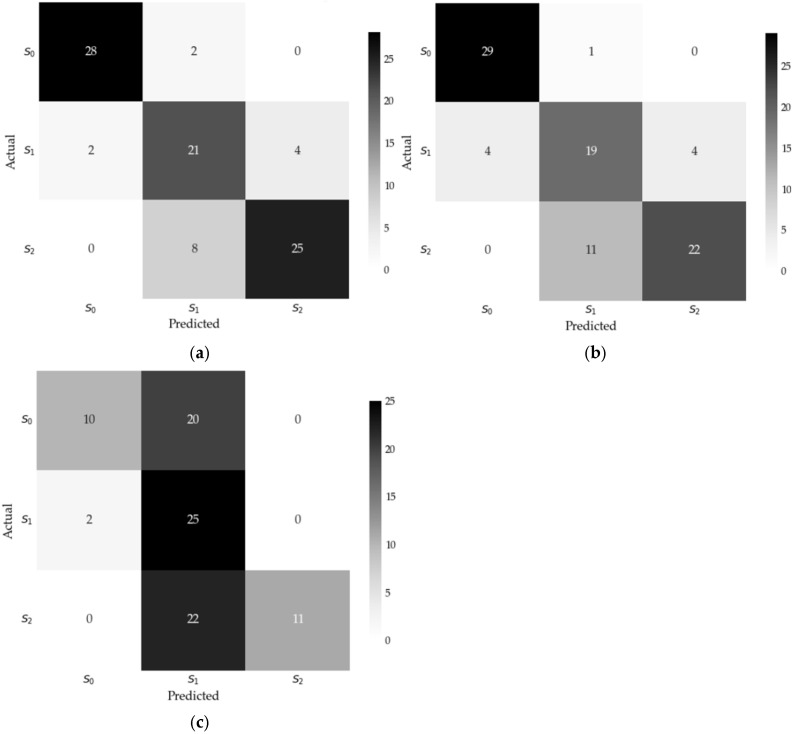
Confusion matrices for each model: (**a**) gradient boosting, (**b**) SVM, (**c**) neural network.

Figure 5 illustrates the performances of the three models in predicting each salinity category specifically on unseen data through their respective confusion matrices. The results highlight that gradient boosting achieves superior performance, with minimal misclassifications across all classes, particularly for S_0_ (low chloride deposition) and S_2_ (high chloride deposition).

In contrast, SVM and neural network exhibit higher error rates, especially for the intermediate category S_1_, where instances are frequently misclassified as either S_0_ or S_2_. Notably, the GB confusion matrix demonstrates a well-balanced distribution of predictions, effectively capturing the patterns within the data and minimizing classification errors across all salinity categories.

### 4.5. Comparative Analysis

**Figure 6:**
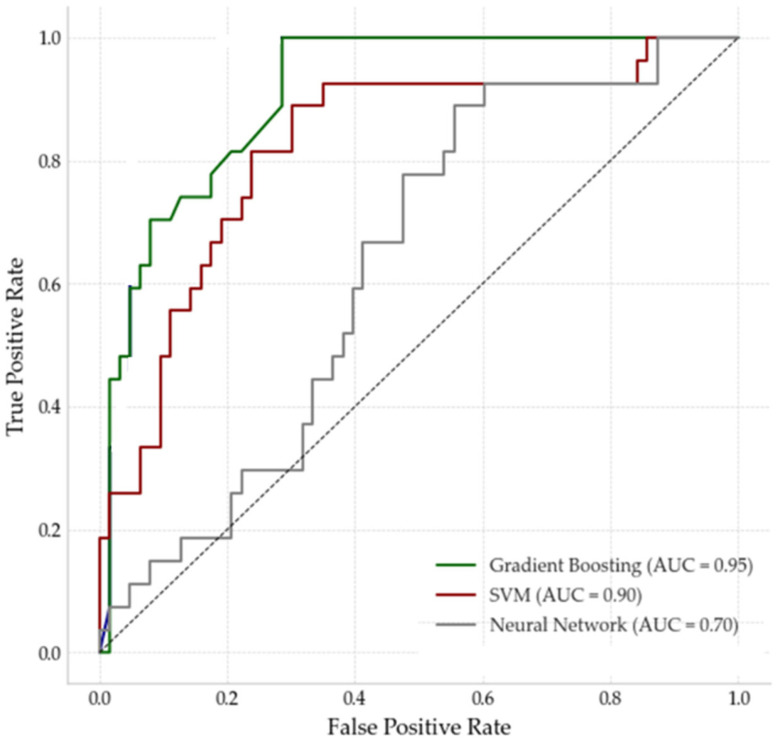
ROC curves and AUC values for each model.

Finally, Figure 6 presents the ROC curves for the three models, illustrating their ability to discriminate between salinity categories. The area under the curve (AUC) values quantify the overall performance of each model. GB achieved the highest AUC (0.95), indicating excellent discriminatory power and minimal false positives across all salinity categories. SVM attained a moderate AUC (0.90), reflecting a good classification capability but slightly lower effectiveness compared to the tree-based models. The neural network exhibited the lowest performance, with an AUC of 0.70, suggesting limited ability to differentiate between salinity levels, particularly in borderline cases.

## 5. Discussion

The findings of this study emphasize the critical role of environmental and exposure-related factors in chloride deposition. The relationship between chloride accumulation and wind exposure, as depicted in Figure 3, highlights the direct influence of prevailing winds on deposition rates. However, while this pattern provides valuable insights, it does not capture the full complexity of interactions between multiple environmental variables, suggesting that additional influencing factors may be at play.

The variable importance analysis further supports the significance of key predictors, with coverage, wind speed, and relative orientation playing a dominant role, in agreement with recent studies on atmospheric corrosion [43]. The choice of a quadratic approach to model distance effects was based on empirical observations, aligning with prior research that demonstrates a nonlinear decrease in chloride deposition as the distance from the sea increases [10]. These results reinforce existing knowledge on atmospheric corrosivity while providing a refined framework early-stage design. Considering that coverage, wind, and relative orientation account for over 30% of the impact, integrating these factors from the outset can help optimize the overall design efficiency.

A major methodological decision in this study was the application of the Synthetic Minority Over-sampling Technique (SMOTE) to address class imbalance. The initial dataset exhibited a strong bias toward class S_1_ (71%), leading to class S_1_ being consistently predicted, limiting the model’s ability to capture minority class patterns. By applying SMOTE, we ensured a more even distribution across classes (Table 3), reducing potential biases and enhancing the reliability of predictions. Similar approaches have been successfully employed in corrosion-related classification tasks, improving model robustness and generalizability [63].

Regarding model performance, gradient boosting outperformed SVM and neural networks by 12.6% and 19.3% in precision and by 12.8% and 27.2% in recall, respectively. This is consistent with previous studies, demonstrating the effectiveness of tree-based models in handling complex, nonlinear relationships in environmental datasets [64]. The superior performance of this model (AUC > 0.95) suggests that it is well-suited for chloride deposition classification, particularly when working with heterogeneous data sources.

SVM and neural network exhibit higher error rates, especially for the intermediate category S_1_, where instances are frequently misclassified as either S_0_ or S_2_. The classification difficulties in class S_1_ stem from the inherent structure of the dataset. Notably, 58% of data points fall within the transition range between classes (±20 mg/cm^2^d), while only 26% of S_1_ instances are in the upper part of the class. This distribution challenges the classification models, as the boundary between S_1_ and the other classes is not well defined. SVM, although useful for certain classification problems, exhibited a limited ability to differentiate moderate chloride deposition levels, a pattern also noted in recent research [10]. Neural networks performed the worst in this study, likely due to the limited size of the dataset. A shallow ANN was used to avoid overfitting, given the limited dataset. Future work will explore deeper or convolutional architectures. Prior studies have suggested that neural networks require larger datasets to generalize effectively [65].

From a practical perspective, the high performance of tree-based models (F1 score > 0.85) suggests they can serve as reliable tools for salinity classification in atmospheric corrosion studies. Their capacity to capture complex interactions makes them particularly valuable for applications requiring predictive assessments of chloride deposition. Additionally, given the lack of standardized predictive models, the most suitable reference remains the descriptive application of ISO 9223 parameters. The use of this model aligns with the ISO 9223:2012 [50] classification system, providing a cost-effective alternative to traditional long-term monitoring methods while reducing uncertainties in the early-stage design of corrosion risk evaluation.

Despite the promising results, this study has several limitations that should be considered. The dataset integrates data from multiple regions, but environmental differences such as climate, pollution, and exposure conditions may affect model generalization. Further validation in diverse geographic locations is necessary to ensure robustness. Additionally, while SMOTE helped address class imbalance, the reliance on synthetic data could introduce biases that do not fully reflect real-world conditions [66], limiting the assessment of true environmental variability.

## 6. Conclusions

This study presents a robust framework for predicting chloride deposition in coastal environments by integrating key geographic and climatic variables with advanced supervised-learning techniques. Unlike traditional approaches, this methodology is based on readily available environmental data, reducing dependency on long-term experimental measurements.

The results highlight the significant role of coverage, wind, and relative orientation in atmospheric corrosion processes, with additional interactions, such as relative humidity and wind, also playing a relevant role. Although wind is not explicitly addressed by ISO standards, it indirectly affects chloride deposition, highlighting its importance in predictive modeling. These findings emphasize the importance of selecting appropriate input variables for predictive models, aligning with previous research on marine corrosion.

Addressing class imbalance significantly enhanced model reliability, increasing the representation of minority classes from 5% to 33% (for class S_0_) and 24% to 33% (for class S_2_), allowing the models to better capture patterns across all chloride deposition levels and reducing the risk of bias towards the majority class (S_1_). This adjustment led to a more reliable and interpretable outcome, as evidenced by improved model performance in all classes.

The analysis of variable importance identified coverage, wind, and relative orientation as key predictors of chloride deposition, contributing 14%, 10%, and 8% of the predictive weight, respectively. These findings emphasize the relevance of these factors in atmospheric corrosion processes, aligning with prior research in marine corrosion.

Tree-based models, specifically gradient boosting, achieved superior predictive performance, with F1 Scores of 0.8673 and AUC values of 0.950. Their robustness and ability to handle complex environmental data make them particularly suitable for this application.

The SVM and neural network models underperformed compared to gradient boosting, with F1 Scores of 0.774 and 0.639. This result suggests that additional tuning or architectural adjustments are necessary for these models to effectively handle complex environmental datasets.

This research demonstrates the effectiveness of tree-based models in predicting chloride deposition using readily available environmental variables. By addressing data imbalance and focusing on relevant predictive factors, the research provides a practical framework for early-stage corrosion risk assessment in coastal regions, supporting the development of more sustainable structural designs.

Future research should focus on expanding the dataset to include a broader range of geographic locations and climatic conditions to further enhance model generalization. To better assess the potential bias introduced by synthetic data, future studies should incorporate external validation on exclusively real-world observations. Additionally, exploring hybrid models that combine tree-based with neural network techniques could offer improvements in predictive accuracy and robustness. Finally, incorporating real-time environmental data through sensor networks would enable continuous training and adaptation of models, ensuring that predictions remain accurate in the face of dynamic atmospheric conditions.

## Figures and Tables

**Table 1:** Summary of modeling approaches for chloride deposition.

Category	Model Type	References
Parametric Regression Models	Exponential Regression	[36]
	Exponential Regression (Wind)	[37]
	Nested Exponential Model	[38]
	Distance Regression with Terrain Correction	[39]
Interpolation Algorithms	Kriging Interpolation	[40]
	Regression + Kriging	[41]
Hybrid Methods	Regression + PEST + Monte Carlo	[42]
Machine Learning Models	Artificial Neural Networks (ANN)	[43]
	Genetic Algorithm-Optimized Quantile Regression Forest (GA-QRF)	[10]

**Table 2:** Categories set by ISO 9223:2012 based on chloride content.

Chloride Deposition Rate [mg/(m^2^·d)]	Level
S_d_ ≤ 3	S_0_
3 < S_d_ ≤ 60	S_1_
60 < S_d_ ≤ 300	S_2_
300 < S_d_ ≤ 1500	S_3_

**Table 3:** Comparison of class distribution before and after balancing.

	Original Situation	Ultimate Situation
S_0_	5%	33%
S_1_	71%	33%
S_2_	24%	33%

**Table 4:** Grid values studied for each model. In bold, the optimized that were chosen.

Gradient Boosting		
Max_Depth	N_Estimators	Learning_Rate
**3**, 5, 10	**50**, 100, 200	0.2, 0.1, **0.01**
**SVM**		
C	Kernel	Gamma
**0.1**, 1, 10	**linear**, rbf	Auto, **scale**
**Neural Networks**		
Hidde_layer_sizes	Activation	Learning_rate_init
(20,), (25,), **(50,)**	**tanh**, relu	**0.001**, 0.01

**Table 5:** Comparative performance metrics for each model.

Model	Precision	Recall
Gradient Boosting	0.869163	0.866667
SVM	0.783341	0.777778
Neural Network	0.734135	0.633333

## Data Availability

The data used to support the findings of this study are available from the corresponding author upon request.
